# Modeling Sialidosis with Neural Precursor Cells Derived from Patient-Derived Induced Pluripotent Stem Cells

**DOI:** 10.3390/ijms22094386

**Published:** 2021-04-22

**Authors:** Binna Seol, Young-Dae Kim, Yee Sook Cho

**Affiliations:** 1 Stem Cell Research Laboratory (SCRL), Immunotherapy Research Center (IRC), Korea Research Institute of Bioscience and Biotechnology (KRIBB), 125 Gwahak-ro, Yuseong-gu, Daejeon 34141, Korea; nana8610@kribb.re.kr (B.S.); kimyoungdae77@gmail.com (Y.-D.K.); 2 Department of Bioscience, KRIBB School, University of Science & Technology, 113 Gwahak-ro, Yuseong-gu, Daejeon 34113, Korea

**Keywords:** induced pluripotent stem cell, sialidosis, lysosomal storage disease, NEU1, neural cell model

## Abstract

Sialidosis, caused by a genetic deficiency of the lysosomal sialidase gene (*NEU1*), is a systemic disease involving various tissues and organs, including the nervous system. Understanding the neurological dysfunction and pathology associated with sialidosis remains a challenge, partially due to the lack of a human model system. In this study, we have generated two types of induced pluripotent stem cells (iPSCs) with sialidosis-specific *NEU1^G227R^* and *NEU1^V275A/R347Q^* mutations (sialidosis-iPSCs), and further differentiated them into neural precursor cells (iNPCs). Characterization of *NEU1^G227R^*- and *NEU1^V275A/R347Q^*- mutated iNPCs derived from sialidosis-iPSCs (sialidosis-iNPCs) validated that sialidosis-iNPCs faithfully recapitulate key disease-specific phenotypes, including reduced NEU1 activity and impaired lysosomal and autophagic function. In particular, these cells showed defective differentiation into oligodendrocytes and astrocytes, while their neuronal differentiation was not notably affected. Importantly, we found that the phenotypic defects of sialidosis-iNPCs, such as impaired differentiation capacity, could be effectively rescued by the induction of autophagy with rapamycin. Our results demonstrate the first use of a sialidosis-iNPC model with *NEU1^G227R^*- and *NEU1^V275A/R347Q^*- mutation(s) to study the neurological defects of sialidosis, particularly those related to a defective autophagy–lysosome pathway, and may help accelerate the development of new drugs and therapeutics to combat sialidosis and other LSDs.

## 1. Introduction

Sialidosis is a lysosomal storage disease (LSD) characterized by an abnormal accumulation of sialylated oligosaccharides and glycolipids resulting from *NEU1* loss of function [1,2]. Sialidosis-causing mutations in the *NEU1* gene, located on chromosome 6p21.3, eliminate the activity or stability of lysosomal sialidase (also known as neuraminidase 1; EC 3.2.1.18), catalyzing the hydrolysis of terminal sialic acid molecules (N-acetylneuraminic acid or NANA) linked to the saccharide chains of glycolipids, glycoproteins, and oligo- and polysaccharides [1,2,3,4]. Lysosomal sialidase forms a multienzyme complex with other lysosomal hydrolases, such as protective protein/cathepsin A (PPCA) and glycosidase β-galactosidase (β-GAL), to promote their canonical degradative activities [5,6]. *NEU1* mutations can cause disruption of the multienzyme complex, ultimately resulting in loss of enzymatic activity [5,7]. None of the other three sialidases, which are distributed in the cytosol (NEU2 neuramidase 2), plasma membrane (NEU3 neuramidase 3), or mitochondrial/lysosomal/intracellular membranes (NEU4 neuramidase 4), appears to compensate for the deficiency of lysosomal sialidase in sialidosis patients [8]. Genetic testing of sialidosis patients has revealed various pathological variants of the *NEU1* gene that are related to a broad range of neurophysiological abnormalities, including visual defects, myoclonus syndrome, cherry-red macular spots, ataxia, hyperreflexia, and/or seizures [9,10]. The clinical symptoms, severity, and penetrance of sialidosis appear to correlate with the types of *NEU1* mutations, which closely reflect the overall biochemical properties of sialidase, including the levels of residual sialidase activity.

Animal models harboring disease-causing mutations are a crucial tool for identifying and determining the common molecular mechanisms and biochemical impact of NEU1 deficiency on sialidosis-related phenotypes and pathogenesis. Preclinical research conducted in Neu1 mutant mouse models revealed defects in virtually all systemic organs, including the nervous system and connective tissues that are similar or consistent with the clinical features of patients with sialidosis [11,12,13,14,15]. A wide range of pathological phenotypes observed in sialidosis mouse models appear to be shared across multiple LSDs [12,13,15]. Homozygous Neu1−/− knockout mouse models display unique pathological phenotypes of sialidosis, but also share some clinical and histopathological features with galactosialidosis caused by a primary defect of the *PPCA* gene (chromosomal locus, 20q13.1) [12]. The brains of Neu1−/− mouse models were shown to exhibit features of Alzheimer’s disease (AD)-related pathology and phenotypes, including increased β-amyloid plaque formation and secretion [13]. Although animal models are very valuable, major disadvantages of these models, including the inability to explicitly define and delineate sialidosis-specific pathological traits from those of other LSDs and the inability to fully recapitulate equivalent human phenotypes, remain. To correctly interpret the possible pathological and clinical contribution of *NEU1* variants observed in sialidosis patients and to develop effective diagnostic and therapeutic strategies, large numbers of highly representative human mutant models are required.

Induced pluripotent stem cell (iPSC) technology, providing a variety of previously inaccessible disease-relevant target cells, opens up a new realm of possibilities for the development of in vitro cell-based disease models. Development of neural lineage cells from disease-specific iPSCs, particularly those derived from neuropathic LSD patients, has contributed to a greater understanding of the neuropathophysiology of LSDs, yielding an effective cellular platform for identifying safer and more effective therapeutic targets [16,17,18,19,20]. Likewise, the development of sialidosis- and tissue-specific cells from iPSCs with sialidosis-associated mutations also offer emerging opportunities to investigate sialidosis at the cellular and molecular level, but they are less studied than other LSDs [21,22,23,24]. However, there have been a limited number of modeling studies for sialidosis. A recent study using sialidosis neurons from two sialidosis-iPSCs with *NEU1* mutations either of p.D176G or of p.P316S revealed functional and molecular abnormalities such as defective presynaptic exocytosis and excessive enhancement of α-amino-3-hydroxyl-5-methyl-4-isoxazole-propionate receptor (AMPAR)-evoked Ca^2+^ influx in sialidosis patients [24]. Nevertheless, the neuropathological features characteristic of sialidosis, involving primary defects in the lysosomal and autophagic machinery, have not been fully elucidated across different *NEU1* mutation types. Large numbers of neurological cell models, covering various types of heterogeneous pathological mutations in *NEU1* gene, are highly desirable to clearly define the neuropathological defects of sialidosis caused by *NEU1* mutation and to develop novel therapeutic strategies.

Previously, we found the novel *NEU1* mutation (p.R347Q) in sialidosis patient fibroblasts showing sialidosis-associated phenotypes [25]. In this study, we aim to establish new sialidosis-specific neural models by producing sialidosis-specific iPSCs and NPCs with *NEU1* mutations (p.V275A/R347Q and p.G227R) to investigate the effects of NEU1 deficiency on autophagy–lysosome pathways and their relevant contribution to neurological defects. Here, we show that sialidosis-iNPCs carried *NEU1^G227R^* or *NEU1^V275A/R347Q^* mutations that closely recapitulate disease-specific phenotypes, such as reduced sialidase activity and autophagy and lysosome dysfunction. Of note, we show that rapamycin treatment effectively rescues sialidosis-related phenotypes such as autophagy and lysosome dysfunction and defective NPC and oligodendrocyte and astrocyte differentiation. We believe that our iPSC models for sialidosis would aid in the elucidation of the molecular pathogenesis as well as in drug discoveries for sialidosis.

## 2. Results

### 2.1. Generation and Characterization of Sialidosis-iPSCs

**Figure 1:**
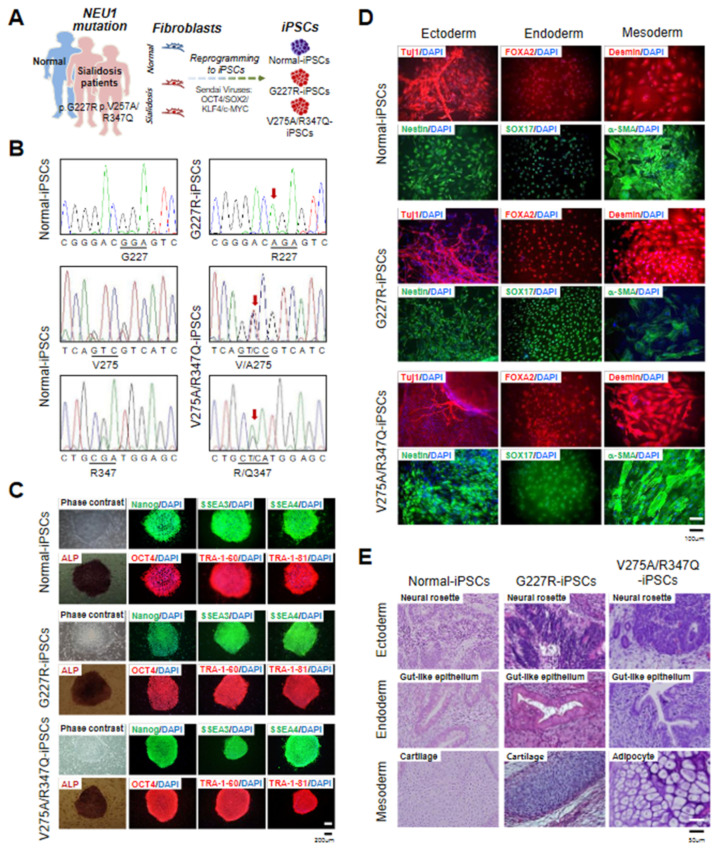
Derivation and characterization of normal and sialidosis-iPSCs. (**A**) Schematic representation of iPSC derivation from fibroblasts. (**B**) DNA sequences of the *NEU1* gene in normal- and sialidosis-iPSCs (G227R- and V275A/R347Q-iPSCs) derived from normal or sialidosis patient fibroblasts carrying the G227R or V275A/R347Q mutations in *NEU1*. (**C**) Representative phase contrast images of iPSCs with or without alkaline phosphatase (ALP) staining and immunohistochemistry images of iPSCs stained for the pluripotency markers NANOG, SSEA3, SSEA4, OCT4, TRA1-60, and TRA-1-81. (Scale bar = 200 μm) (**D**) Representative immunohistochemistry images of differentiated iPSCs stained for ectoderm (TUJ1 and NESTIN), endoderm (FOXA2 and SOX17), and mesoderm (DESMIN and α-SMA) markers. Nuclei were counterstained with DAPI (blue). (Scale bar = 100 μm) (**E**) Representative images of hematoxylin and eosin (H&E)-stained teratoma sections showing the three germ layers: ectoderm (top panel), endoderm (middle panel), and mesoderm (bottom panel). (Scale bar = 50 μm).

To generate sialidosis-specific iPSC models, fibroblasts from two sialidosis patients with different mutations [p.G227R and p.V275A/R347Q] in the *NEU1* gene were reprogrammed into iPSCs using Sendai virus (SeV) vectors encoding for OCT4, SOX2, KLF4, and C-MYC (Figure 1A). Normal-iPSCs generated from human foreskin fibroblasts (ATCC-CRL2097) were used as the control. Normal- and sialidosis-iPSCs (G227R-iPSCs and V275A/R347Q-iPSCs) were manually picked based on an undifferentiated ESC-like morphology between day 21 and day 28 after reprogramming and expanded for further characterization. The disease-causing *NEU1^G227R^*- and *NEU1^V275A/R347Q^*-mutations determined in the two different parent fibroblasts used for reprogramming (Figure S1) [25] were confirmed in the established G227R-iPSCs and V275A/R347Q-iPSCs, respectively (Figure 1B). All the iPSC lines (normal-iPSCs, G227R-iPSCs, and V275A/R347Q-iPSCs) exhibited an undifferentiated hESC-like morphology, similar alkaline phosphatase (ALP) activity, and high expression of pluripotency markers (NANOG, OCT4, SSEA3, SSEA4, TRA-1-60, and TRA-1-81) (Figure 1C). The pluripotency of normal- and sialidosis-iPSCs was further confirmed by in vitro (Figure 1D) and in vivo trilineage differentiation into ectoderm, endoderm, and mesoderm (Figure 1E). Standard G-banding analysis showed that a normal karyotype (46, XY) was maintained in the normal- and sialidosis-iPSCs (Figure S2). Short tandem repeat (STR) analysis confirmed that all the iPSC lines were derived from their corresponding parental fibroblasts (Table S1). Our results showed that normal- and sialidosis-iPSCs (G227R-iPSCs and V275A/R347Q-iPSCs) were indistinguishable across lines by gross pluripotency assays.

### 2.2. Generation and Characterization of Sialidosis-iNPCs

**Figure 2:**
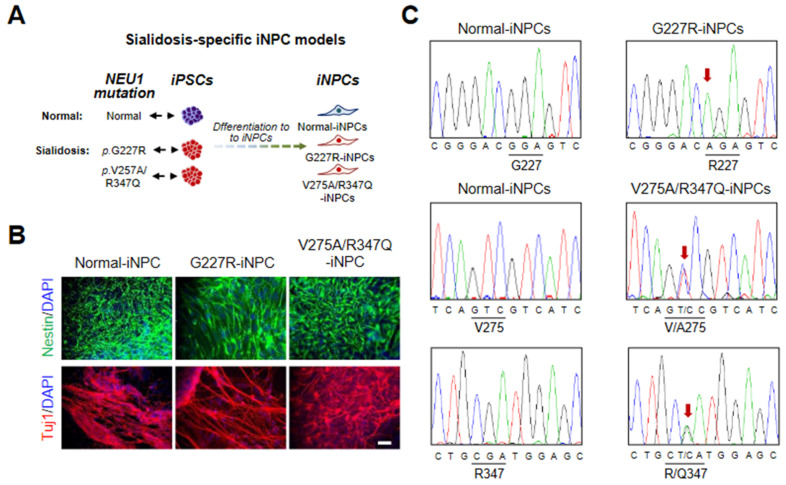
Derivation and characterization of normal and sialidosis-iNPCs. (**A**) Schematic representation of iNPC derivation from iPSCs. (**B**) Representative immunohistochemistry images of normal- and sialidosis-iNPCs (G227R- and V275A/R347Q-iNPCs) stained for the NPC markers Nestin and Tuj1. Nuclei were counterstained with DAPI (blue). (Scale bar = 50 μm) (**C**) DNA sequences of the *NEU1* gene in normal- and sialidosis-iNPCs derived from normal or sialidosis-iPSCs carrying the G227R or V275A/R347Q mutation in *NEU1*.

Subsequently, normal- and sialidosis-iPSCs (G227R-iPSCs and V275A/R347Q-iPSCs) were differentiated into normal- and sialidosis-iNPCs (G227R-iNPCs and V275A/R347Q-iNPCs) through an embryoid body (EB) intermediate using neural differentiation medium (Figure 2A,B). We confirmed that the *NEU1^G227R^* and *NEU1^V275A/R347Q^* mutations were maintained in the established G227R-iNPC and V275A/R347Q-iNPC, respectively (Figure 2C).

**Figure 3:**
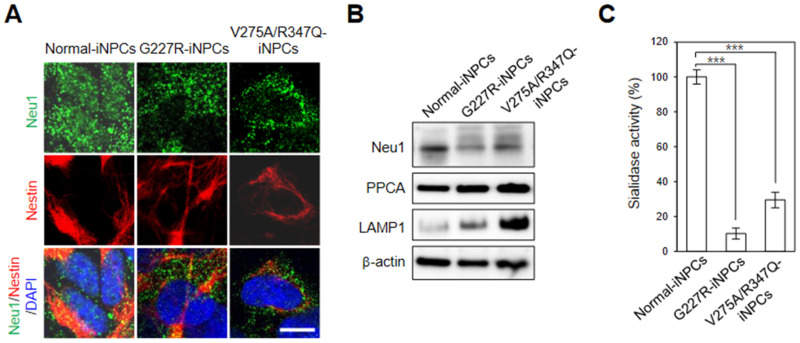
Disease-specific phenotypes of sialidosis-iNPCs. (**A**) Representative immunohistochemistry images of normal- and sialidosis-iNPCs (G227R- and V275A/R347Q-iNPCs) stained for NEU1 and the NPC marker Nestin. Nuclei were counterstained with DAPI (blue). (Scale bar = 10 μm) (**B**) Western blot analysis of NEU1, PPCA, and LAMP1 expression in normal- and sialidosis-iNPCs. β-actin was used as the loading control. (**C**) Sialidase activity of normal- and sialidosis-iNPCs. The data are the means ± SDs of at least 3 independent experiments. *** *p* < 0.001, compared to control normal-iNPCs (*t*-test).

Immunofluorescence analysis of the sialidosis-iNPCs with an anti-NEU1 antibody showed a similar punctate endo-/lysosomal staining pattern as seen in the normal-iNPCs, while the expression of NEU1 in the sialidosis-iNPCs (G227R-iNPCs and V275A/R347Q-iNPCs) had a more perinuclear distribution compared with that of the normal-iNPCs (Figure 3A). Notably, lower protein levels of NEU1 were detected in the sialidosis-iNPCs (G227R-iNPCs and V275A/R347Q-iNPCs) compared to normal-iNPCs (Figure 3B). Accordingly, we observed decreased sialidase activity in the sialidosis-iNPCs [approximately 10% (G227R-NPCs) and 16% (V275A/R347Q-NPCs) of normal control] (Figure 3C). The activity and stability of Neu1 is known to be exclusively influenced by association with other lysosomal enzymes such as protective protein/cathepsin A (PPCA) and β-galactosidase [5,6]. Western blot analysis also showed that the protein expression levels of PPCA were not notably different between the normal- and sialidosis-iNPCs (Figure 3B), suggesting that *NEU1^G227R^* and *NEU1^V275A/R347Q^* mutation-mediated loss of NEU1 activity is not correlated with PPCA expression.

### 2.3. NEU1 Deficiency-Associated Autophagy–Lysosomal Dysfunction in Sialidosis-iNPCs

Sialidase NEU1, a known negative regulator of lysosomal exocytosis, is responsible for the processing of sialic acids on lysosomal-associated membrane protein-1 (LAMP-1), a lysosomal exocytosis effector [26]. Similar to other NEU1-deficient models [26,27], the expression of LAMP1 was markedly increased in the sialidosis-iNPCs (G227R-iNPCs and V275A/R347Q-iNPCs), compared with the normal-iNPCs (Figure 3B). These results suggest that NEU1 deficiency due to *NEU1* G227R and V275A/R347Q mutations may result in impaired lysosomal exocytosis in sialidosis-iNPCs and also demonstrate that the features of neuronal and biochemical defects associated with *NEU1^G227R^* and *NEU1^V275A/R347Q^* mutations can be effectively recapitulated in our sialidosis-iNPC models.

**Figure 4:**
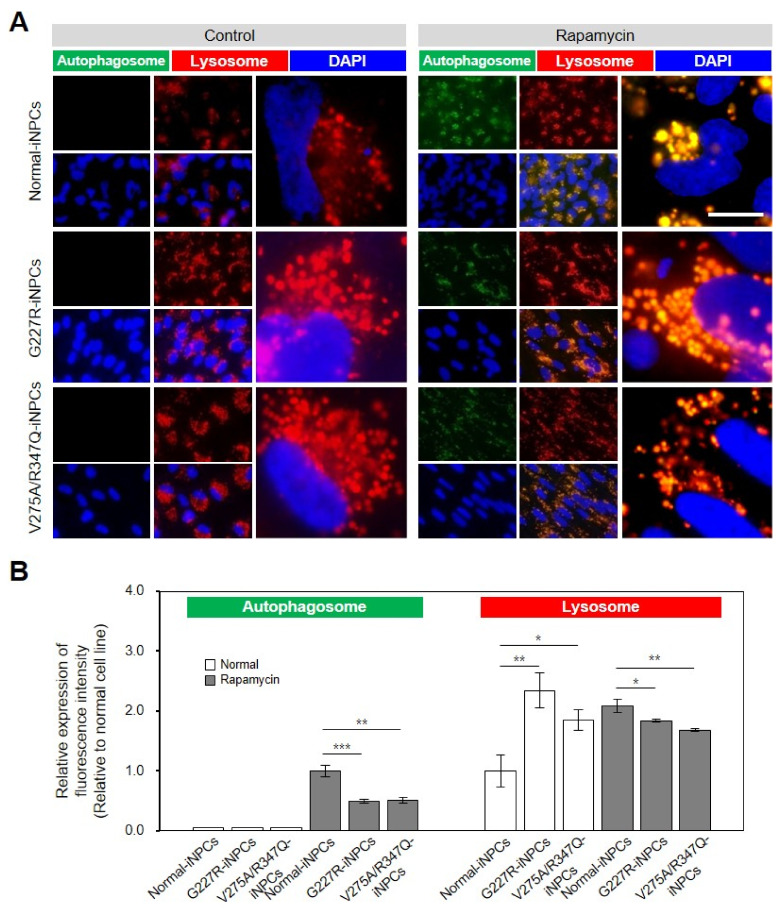
Lysosomal and autophagic dysfunctions in sialidosis-iNPCs. (**A**) Representative images show the LYSO-ID-stained lysosomal (red) and CYTO-ID-stained autophagosomal (green) compartments in normal- and sialidosis-iNPCs cultured in the absence (control, left panel) or presence of rapamycin (500 ng/mL, right panel). Nuclei were counterstained with DAPI (blue). (Scale bar = 10 μm) (**B**) Quantification of the autophagosomal and lysosomal compartments from Figure 4A images to measure the relative expression of fluorescence intensity. The data are the means ± SDs of at least 3 independent experiments. * *p* < 0.05, ** *p* < 0.01, and *** *p* < 0.001, compared to control normal-iNPCs (*t*-test).

A phenotypic LYSO-ID staining assay revealed a comparable number and size of lysosomes/cells in the normal- and sialidosis-iNPCs (G227R-iNPCs and V275A/R347Q-iNPCs). The sialidosis-iNPCs (G227R-iNPCs and V275A/R347Q-iNPCs) showed characteristic enlarged lysosomes compared to the normal-iNPCs (Figure 4, red). The sialidosis-iNPCs (G227R-iNPCs and V275A/R347Q-iNPCs) exhibited brightly stained large, perinuclear-clustered, and scattered lysosomes in the peripheral region, while the normal-iNPCs showed lightly stained, smaller lysosomal vesicles with less perinuclear clustering (Figure 4A, red). Increasingly, studies have demonstrated that impaired lysosomal function observed in various LSDs is associated with autophagy impairment [22,28,29,30]. Unstimulated basal levels of autophagic vacuoles were not detectable in either normal- or sialidosis-iNPCs by the CYTO-ID autophagy detection assay; thus, we evaluated the impact of rapamycin, an mTOR-dependent autophagy inducer [31], in the sialidosis-iNPCs and compared them to normal-iNPCs (Figure 4A,B, green). Rapamycin treatment effectively enhanced the appearance of autophagic vacuoles in both the normal- and sialidosis-iNPCs (Figure 4A,B, green). Notably, rapamycin was less effective in inducing autophagy in the sialidosis-iNPCs than in the normal-iNPCs, which confirmed the Neu1 deficiency-associated inhibition of the autophagic flux in sialidosis-iNPCs (Figure 4A,B). Taken together, these results characterize an impairment of the autophagy–lysosomal pathway in the sialidosis-iNPC model.

### 2.4. Rapamycin Partly Restores Impaired Function of Sialidosis-iNPCs

**Figure 5:**
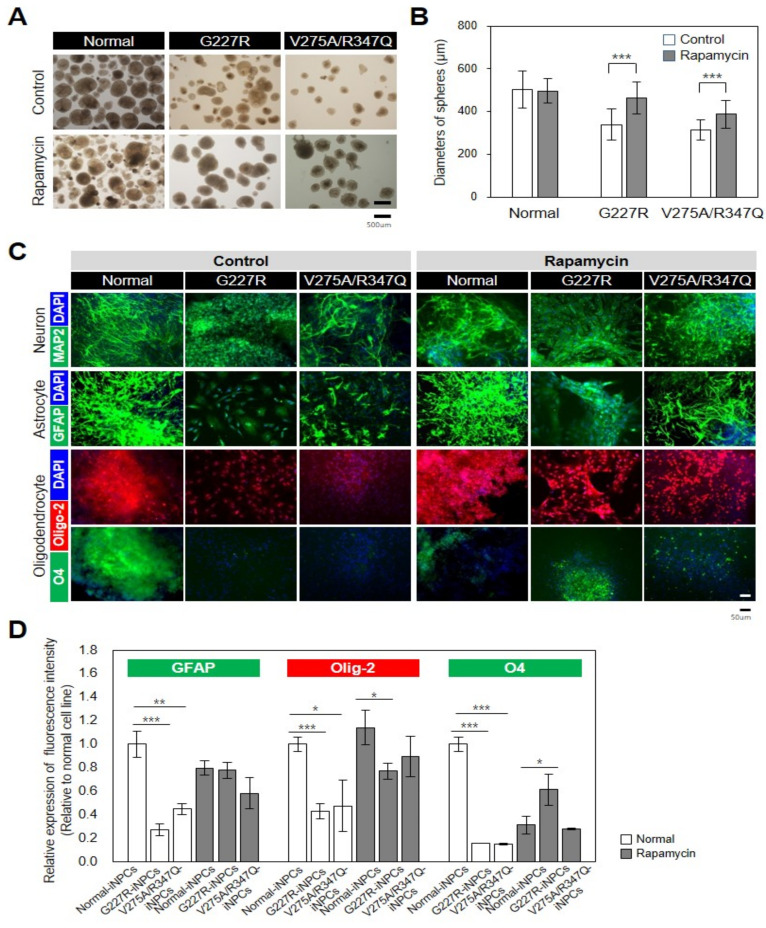
Rescue of disease-associated defects in sialidosis-iNPCs. (**A**) Representative images showing morphological differences between normal- and sialidosis-iNPC spheres (G227R and V275A/R347Q) generated in the absence or presence of 0.1 nM rapamycin treatment. (**B**) Quantification of the average diameters (μm) of normal- and sialidosis-iNPC spheres (G227R and V275A/R347Q) shown in A. The data are the means ± SDs of three independent experiments. *** *p* < 0.001, compared to untreated control (*t*-test). (**C**) Representative immunohistochemistry images of normal- and sialidosis-iNPCs (G227R and V275A/R347Q) differentiated into neurons, astrocytes, and oligodendrocytes and stained for the indicated lineage-specific markers. (Scale bar = 50 μm) (**D**) Quantification of the differentiation potential from Figure 5C images to measure the relative expression of fluorescence intensity. The data are the means ± SDs of at least 3 independent experiments. * *p* < 0.05, ** *p* < 0.01, and *** *p* < 0.001, compared to control normal cell line (*t*-test).

As the sialidosis-iPSCs, relative to the normal-iPSCs, were found to be less efficient in the production of NPCs (Figure 5A,B), we next evaluated whether the differentiation capability of the sialidosis-iPSCs into NPCs could be restored by activation of autophagy with rapamycin. Notably, rapamycin treatment effectively increased the production yields of the sialidosis-iNPCs, as determined by increases in spherical NPC size and numbers (Figure 5A,B). While the normal- and sialidosis-iNPCs did not differ in their capabilities to differentiate into neurons, the sialidosis-iNPCs showed reduced differentiation towards oligodendrocytes and astrocytes (Figure 5C,D). Significantly, the defective differentiation potential of the sialidosis-iNPCs, especially into oligodendrocytes and astrocytes, was notably rescued by rapamycin treatment (Figure 5C,D). These observations demonstrate that dysfunction of sialidosis-iPSCs and -iNPCs can be rescued, at least in part, by autophagy activation. In addition, the reversal of the phenotypic defects of sialidosis-iNPCs with rapamycin can provide future opportunities for therapeutic strategies.

## 3. Discussion

**Figure 6:**
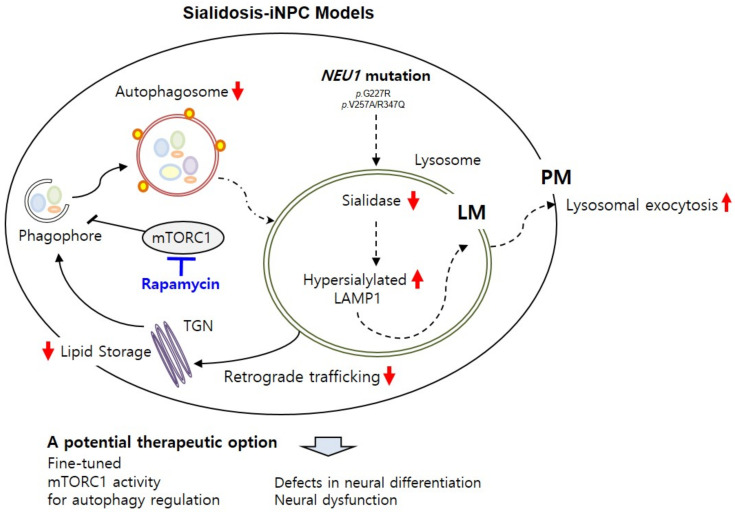
Schematic summary of sialidosis-specific neurological impairments characterized in sialidosis-iNPCs. The sialidosis-iNPC model reveals molecular defects in sialidosis, such as *NEU1* mutation, deficient sialidase activity, and increased LAMP1 expression. Deficient NEU1 enzyme activity caused by genetic mutations in the *NEU1* gene results in impaired autophagy and lysosomal pathways in sialidosis-iNPCs. Induction of autophagy by rapamycin effectively rescues sialidosis-related defects in sialidosis-iNPCs, suggesting an important role of the autophagy–lysosome pathway in neurological abnormalities associated with sialidosis.

Here, we present new sialidosis-iNPC models carrying *NEU1^G227R^* or *NEU1^V275A/R347Q^* mutations generated from the fibroblasts of two independent patients with p.G227R (c.677G > A) and p.V275A (c.824T > C)/p.R347Q (c.1040G > A) mutations [25,32]. All *NEU1^G227R^*- or *NEU1^V275A/R347Q^*-mutated iNPC models showed NEU1 deficiency-mediated autophagy and lysosomal dysfunction and loss of differentiation capacity, which were closely related to neurological dysfunction that occurred because of sialidosis (Figure 6).

Although beneficial effects of various therapeutic approaches, including enzyme replacement therapy and small molecule drugs, have been demonstrated in various LSDs, such as mucopolysaccharidosis type II (MPSII) [33], Wolman disease (WD) [34], Tay-Sachs disease (TSD) [35], and Niemann-Pick type C1 disease (NPC1) [36], there is still no cure for most LSDs. Analysis of neural cell models derived from iPSCs from patients with LSDs including Niemann-Pick type C1 disease (NPC1) [36,37], GM1 gangliosidosis [38], TSD [35,39], mucopolysaccharidosis type I (MPSI) [40], MPS type II (MPSII) [33,41], MPS VII [42], and WD [34], has yielded important insights into causative genes and the pathogenesis associated with the neurological abnormalities that are characteristic of sialidosis. Sialidosis-iPSCs, harboring both sialidosis-associated mutations and patient-specific genetic background, offer emerging opportunities to investigate sialidosis at the cellular and molecular level. However, only four reports have been published on sialidosis-iPSC models from patients with different *NEU1* mutations [21,22,23,24]. Two reports demonstrated the establishment of sialidosis-iPSC models with *NEU1* mutations c.649G > A/644 T > C, c.1109A > G/c.1109A > G [43], c.1195_1200dup/c.679G > A [23,32], or c.544 A > G transition and 667–679 deletion from sialidosis patient fibroblasts [21]. A review of the literature revealed no additional reports of using those models for the production of an adequate tissue-specific disease model, but they can be a potential source of it. As a tissue-specific disease model, retinal pigment epithelial cells (RPEs) were produced from a salidosis-iPSC model with *NEU1* mutations c.239C > T and c.403G > A [44] and thereby revealed the NEU1 deficiency-associated impaired autophagy in sialidosis-specific RPEs [22]. Recently, characterization of sialidosis neurons derived from two sialidosis-iPSCs carrying *NEU1^D176G^* or *NEU1^P316S^* has revealed the functional and molecular abnormalities such as defective presynaptic exocytosis and excessive enhancement of AMPA-evoked Ca2^+^ influx in sialidosis [24]. 

Similarly to sialidosis-NPCs carrying *NEU1^D176G^* or *NEU1^P316S^* [21], our sialidosis-iNPC models carrying *NEU1^G227R^* or *NEU1^V275A/R347Q^* mutations revealed correlations of *NEU1* mutations with sialidosis-specific molecular defects and disease phenotypes, which are specifically associated with abnormal lysosomal dynamics. *NEU1^G227R^*- and *NEU1^V275A/R347Q^*-iNPCs showed 10% and 29% residual activity respectively, compared to the normal control cells. These results correspond to those determined in the parental patient fibroblasts [25] and other pathological mutations identified in the *NEU1* gene [4,24,32]. The expression levels of PPCA, a major constituent of the multienzyme lysosomal complex, were not notably changed in *NEU1^G227R^*- and *NEU1^V275A/R347Q^*-iNPCs compared to normal control cells. However, there is the possibility that *NEU1^G227R^* and *NEU1^V275A/R347Q^* mutations may affect the NEU1-PPCA interaction essential for the correct trafficking of NEU1 to lysosomes, where the NEU1 enzyme is processed into its mature, active form [7,43,45]. As NEU1 acts as a negative regulator of lysosomal exocytosis mediating cellular clearance by processing its substrate LAMP1 sialic acids, NEU1 deficiency-associated accumulation of over-sialylated LAMP1 at the lysosomal membrane appears to be responsible for excessive lysosomal exocytosis [13,26]. Consistent with previous observations in sialidosis-NPCs carrying *NEU1^D176G^* or *NEU1^P316^*^S^ [21], loss of NEU1 enzyme activity in *NEU1^G227R^*- and *NEU1^V275A/R347Q^*-iNPCs is coupled with increased expression of LAMP1, which is potentially suggestive of impaired lysosomal exocytosis in *NEU1^G227R^*- and *NEU1^V275A/R347Q^*-iNPCs, but the detailed molecular machinery and regulatory mechanisms require further investigation.

The role of autophagy malfunction has been extended to LSDs [28,29] but has not been clearly investigated in sialidosis in terms of neurological dysfunction. Our observations provide the first evidence that NEU1 deficiency alters not only lysosomal dynamics but also autophagic activity in *NEU1^G227R^*- and *NEU1^V275A/R347Q^*-iNPCs, whereas we could not exclude the possibility of retrograde trafficking pathway involvement (Figure 6) [46]. Although *NEU1^G227R^*- and *NEU1^V275A/R347Q^*-iNPCs were found to be less sensitive to rapamycin than normal-iNPCs, rapamycin treatment effectively recovered autophagic flux in *NEU1^G227R^*- and *NEU1^V275A/R347Q^*-iNPCs. Significantly, autophagy activation with rapamycin effectively ameliorated the accumulation of enlarged lysosomes in *NEU1^G227R^*- and *NEU1^V275A/R347Q^*-iNPCs, suggesting a connection between impaired autophagic flux and lysosomal dysfunction. Importantly, the defective differentiation potential of both *NEU1^G227R^*- and *NEU1^V275A/R347Q^*-iPSCs into iNPCs and *NEU1^G227R^*- and *NEU1^V275A/R347Q^*-iNSCs into oligodendrocytes and astrocytes was effectively rescued by rapamycin treatment, demonstrating the potential of targeting these glial cells for therapeutic intervention. Consistently, many studies indicate the contribution of impaired astrocytes, triggered by autophagic/lysosomal dysfunction, to driving neuronal dysfunction in other LSDs, such Alzheimer’s disease, Parkinson’s disease, and Huntington disease, and highlight the potential of targeting these cells as a beneficial therapeutic strategy for LSDs [47,48,49,50]. However, it still remains unexplored in the context of sialidosis neuropathogenesis. Moving forward, it would be important to address whether and how glial cells play a role on sialidosis progression and pathobiology and to determine their impact on neuronal circuits and networks.

In pursuit of more accurate and reproducible modeling of sialidosis, multiple iPSC models, covering various types of heterogeneous pathological mutations in *NEU1* gene, a variety of donors, and differentiation variation, are highly desirable for comparative studies, but still are not available. Our findings show that sialidosis-iNPCs with G227R or V275A mutations share some common pathological and biochemical features with other mutations types of sialidosis, and the utility of our *NEU1^G227R^*- and *NEU1^V275A/R347Q^*-iNPCs can be expanded to comparative and interdisciplinary research with other sialidosis models to better understand phenotype–genotype correlations and common and distinct pathological mechanisms. Recently, gene editing technology, such as the easily accessible CRISPR/Cas9 system, has been applied for therapeutic purposes and disease modeling of LSDs by correcting or inducing disease-causing mutations [51]. Together with patient-derived sialidosis-iPSC models, with the use of CRISPR gene-edited sialidosis-iPSC models, will allow us to gain a deeper understanding of the molecular mechanisms underlying the impact of disease-causing mutations, and new therapeutic approaches.

In conclusion, our new *NEU1^G227R^*- and *NEU1^V275A/R347Q^*-iNPC models described here closely mimic the altered autophagy and lysosomal dysfunction associated with neurological dysfunction in sialidosis. The findings of this study also highlight the importance of understanding the mechanisms that control the autophagy–lysosome pathways for the development of effective therapies for sialidosis.

## 4. Materials and Methods

### 4.1. Human Fibroblasts

Sialidosis patient fibroblasts with *NEU1^G227R^* (GM02685) or *NEU1^R347Q/V275A^* (GM02921) mutations [25] were obtained from the Coriell Cell Repositories (Camden, NJ, USA: http://ccr.coriell.org, last access date 19th of April 2021). Human newborn foreskin fibroblasts (CRL2097) obtained from ATCC (Manassas, VA, USA) were used as a normal control. Cells were cultured in α-modified Eagle’s minimum essential medium (α-MEM, Thermo Fisher Scientific, Waltham, MA, USA) supplemented with 15% fetal bovine serum (FBS, Invitrogen, Carlsbad, CA, USA), 1× nonessential amino acids (NEAA, Thermo Fisher Scientific), 1% sodium pyruvate (Thermo Fisher Scientific), and 1% penicillin/streptomycin (P/S) (Sigma, St. Louis, MO, USA).

### 4.2. cDNA Sequencing

Total RNA was extracted from fibroblasts, iPSCs, and iNPCs using RNiso Plus reagent (Takara, Shiga, Japan) and was reverse transcribed using the Superscript III first-strand synthesis system (Invitrogen) according to the manufacturers’ instructions. Full-length NEU1 cDNAs were amplified by PCR and sequenced as previously described [25].

### 4.3. Normal- and Sialidosis-iPSCs

Integration-free normal- and sialidosis-iPSCs (*NEU1^G227R^*- and *NEU1^V275A/R347Q^*-iPSCs) were reprogrammed from normal (CRL2097) and sialidosis patient fibroblasts (GM02685 and GM02921), using the CytoTune™-iPS 2.0 Sendai reprogramming kit (Thermo Fisher Scientific) according to the manufacturer’s instructions. The established iPSCs were routinely cultured on Matrigel (BD Biosciences, San Diego, CA, USA)-coated plates in mTeSR1 medium (STEMCELL Technologies, Vancouver, Canada) at 37 °C in 5% CO_2_ and passaged within 4–5 days of culture at a 1:3 ratio using 1 mg/mL dispase (STEMCELL Technologies).

### 4.4. Alkaline Phosphatase (ALP) Analysis

Normal- and sialidosis-iPSC colonies were fixed in a citrate-acetone-formaldehyde solution and stained for ALP activity using an ALP detection kit (Sigma). Cell images were captured using an Olympus microscope (IX51, Olympus, Shinjuku-ku, Tokyo, Japan).

### 4.5. In Vitro EB Formation and Trilineage Differentiation

The iPSC colonies cultured in feeder-free conditions were dissociated with 1 mg/mL collagenase IV. Embryoid body (EB) formation was carried out in suspension culture by culturing of iPSC aggregates with EB medium consisting of knockout Dulbecco’s modified Eagle’s medium (DMEM, Thermo Fisher Scientific), 10% KnockOut serum replacement (KSR), 1× NEAA, 0.1 mM β-mercaptoethanol, and 1 mM L-glutamine in ultralow attachment plates (Corning, Corning, NY, USA) for 5 days. For spontaneous trilineage differentiation, the EB aggregates were transferred to gelatin-coated plates, cultured for an additional 10 days, and analyzed for trilineage marker expression by immunocytochemistry. The medium was changed every other day. The antibodies utilized are summarized in Supplementary Table S2.

### 4.6. G-Banding Karyotyping and Short Tandem Repeat (STR) Profiling

To monitor genomic stability, after more than 20 passages in feeder-free medium, iPSCs were processed for standard chromosomal G-band analysis by GenDix, Inc. (Seoul, Korea). A representative image was obtained by ChIPS-Karyo (Chromosome Image Processing System, GenDix, Inc.). STR profiling of the original sialidosis patient cells (6 passages) and iPSCs (15 passages) was performed by HumanPass, Inc. (Seoul, Korea) to confirm that all the iPSCs had the same origin as patient fibroblasts. The absence of mycoplasma contamination was regularly checked by a polymerase chain reaction (PCR)-based method using an EZ-PCR^TM^ Mycoplasma detection kit (Biological Industries, Beit-Haemek, Israel).

### 4.7. Teratoma Analysis

iPSCs (1 × 10^6^) mixed with 50% v/v Matrigel were injected subcutaneously into the dorsolateral area of 4-week-old CAnN.Cg-Foxn1 nu/CrljOri mice (Orient Bio Inc., Seoul, Korea). Ten weeks after injection, the tumor tissues were dissected and fixed in 4% paraformaldehyde. Paraffin-embedded tissue sections were then stained with hematoxylin and eosin (H&E) solution (Sigma) to analyze teratoma composition.

### 4.8. Immunocytochemistry

Cells were fixed with 4% formaldehyde (Sigma) for 10 min, permeabilized with 0.1% Triton X-100 for 30 min, and blocked with 3% bovine serum albumin (BSA) in phosphate-buffered saline (PBS) for 2 h. The cells were then incubated overnight at 4 °C with primary antibodies diluted in blocking solution. After washing three times with 0.1% Tween-20-containing PBS at room temperature (RT), the cells were further incubated with fluorescence-conjugated secondary antibodies for 2 h and then counterstained with 300 nM 4′, 6-diamidino-2-phenylindole (DAPI, Invitrogen) at RT for 15–30 min. The images were obtained by using an Axio VertA.1 microscope (Carl Zeiss, Oberkochen, Germany) or an Olympus microscope (Olympus, Japan). The antibodies utilized are summarized in Supplementary Table S2.

### 4.9. Normal and Sialidosis-iNPCs

For NPC differentiation, floating EBs from normal- and sialidosis-iPSCs (*NEU1^G227R^*- and *NEU1^V275A/R347Q^*-iPSCs) were attached to Matrigel-coated plates and cultured in neural differentiation medium (NDM) consisting of DMEM/F12 (Thermo Fisher Scientific), 1× N2/B27 (Invitrogen), 20 ng/mL basic fibroblast growth factor, 20 ng/mL epidermal growth factor (Invitrogen, USA), and 10 ng/mL leukemia inhibitory factor (Sigma) for 14 days. Differentiated NPCs were subcultured every week using a Mcllwain^TM^ tissue chopper with a diameter of 200 μm (Mickle Engineering, Gomshall, Surrey, UK), and the medium was refreshed every other day. For proliferation assays, 6 NPC aggregates x per condition were seeded into 96-well plates and cultured in NDM for 14 days. The established NPC spheres were visualized under an Olympus microscope and photographed. The average diameter (μm) of 14–24 NPC spheres was measured using ImageJ software (NIH, Bethesda, MA, USA). For terminal differentiation, NPCs (1 × 10^6^ cells/mL) dissociated with 1× Accutase solution (Invitrogen) were seeded onto Matrigel-coated plates and cultured in neuron [a 1:1 mixture of Neurobasal^TM^ medium (Thermo Fisher Scientific) and DMEM/F12 supplemented with 1× B27 (Thermo Fisher Scientific), 1× GlutaMax (Thermo Fisher Scientific), 1×N2/B27 (Invitrogen), 1 μM retinoic acid (RA, Sigma), 20 μg/mL ascorbic acid (AA) (Sigma), 10 ng/mL brain-derived neurotrophic factor (BDNF, PeproTech, Rocky Hill, NJ, USA), 10 ng/mL glial-cell-line-derived neurotrophic factor (GDNF; PeproTech), and 5 μM forskolin (Sigma)] or astrocyte [a high glucose DMEM supplemented with 1× N2 (Thermo Fisher Scientific), 1× GlutaMax, and 1% FBS] differentiation medium for 3–4 weeks. For oligodendrocyte differentiation, NPCs seeded on Matrigel-coated plates were cultured in differentiation medium I [DMEM/F12 supplemented with 1× N2/B27 (without vitamin A), 1× NEAA, 2 mg/mL heparin (Sigma), 1 μM RA, and 1 μM sonic hedgehog (Peprotech) for 2 weeks] and then in differentiation medium II [DMEM/F12 supplemented with 1× N2/B27 (without vitamin A), 1× NEAA, 601 ng/mL triiodothyronine (T3), 1 μM cyclic AMP (Sigma), 20 ng/mL platelet-derived growth factor (Peprotech), 10 ng/mL insulin-like growth factor (R&D Systems, Minneapolis, MN, USA), and 10 ng/mL neurotrophin-3 (Peprotech)] for 1–2 weeks. The differentiation of iPSCs and iNPCs into target differentiated cells was further performed in the presence of 0.1 nM rapamycin to test whether autophagy affects the differentiation potential and function of the cells.

### 4.10. Sialidase Assay

Cells harvested in assay buffer (20 mM citrate pH 4.5, 60 mM NaCl, and 1 mM MgCl_2_) were lysed by sonication. The cell lysate (2 μg) was added to 100 μL assay buffer containing 0.1 mM 2′-(4-methylumbelliferyl)-α-D-N-acetylneuraminic acid (4-MUNANA, Sigma) substrate and incubated at 37 °C for 4 h. The reactions were terminated by adding 50 μL of 0.1 M Glycine-NaOH (pH 10.5) buffer. The fluorescence signal of the resulting 4-lumbelliferone (4-MU) was determined with excitation and emission at 365 nm and 450 nm, respectively, by using a SpectraMax^®^ M3 Multi-Mode Microplate Reader (Molecular Devices, Sunnyvale, CA, USA).

### 4.11. Western Blot

Cell lysates (20 μg protein) prepared in RIPA buffer containing 50 mM Tris–HCl, pH 8.0, 150 mM sodium chloride, 1% Triton X-100, 0.5% deoxycholic acid, and 0.1% SDS were separated by 4–20% SDS-PAGE and blotted onto PVDF membranes. After blocking in blocking buffer containing 5% skim milk and 0.1% Tween-20 in Tri-buffered saline (TBST) for 2 h at RT, the blots were washed three times for 5 min with TBST and incubated with primary antibodies diluted in 3% skim milk in TBST. After washing the membrane three times with TBST for 5 min, the horseradish peroxidase (HRP)-conjugated secondary antibodies against each primary antibody species was diluted in 3% skim milk in TBST and incubated for 2 h at RT. The bands were detected using an ECL substrate kit (Thermo Fisher Scientific) and normalized to the density of the loading control, β-actin. The antibodies utilized are summarized in Supplementary Table S2.

### 4.12. Autophagy and Lysosome Staining

The iNPCs were seeded on 18-mm cover-glass bottom dishes and untreated or treated with 500 ng/mL rapamycin for overnight. After the treatments, the cells were stained with a CYTO-ID^®^ autophagy detection kit (Enzo, Farmingdale, NY, USA) and LYSO-ID^®^ Red detection kit (ENZ-51005-0100, Enzo) according to the manufacturer’s instructions. Images were acquired by an Axio VertA.1 microscope (Carl Zeiss).

### 4.13. Statistical Analysis

The results are presented as the mean ± SE. Student’s unpaired *t*-test was used for statistical evaluation, and * *p* < 0.05, ** *p* < 0.01, and *** *p* < 0.001 were considered significant. Image results were quantified by ImageJ software (NIH, Bethesda, MD, USA).

## Data Availability

Data is contained within the article or Supplementary Materials.

## Supplementary Materials

The following are available online at https://www.mdpi.com/article/10.3390/ijms22094386/s1.
