# Supplementary material for: Modeling Sialidosis with Neural Precursor Cells Derived from Patient-Derived Induced Pluripotent Stem Cells
Source: Int J Mol Sci. 2021 Apr 22;22(9):4386. doi: 10.3390/ijms22094386 (PMC8122832; doi:10.3390/ijms22094386)
Supplement: Supplementary file 1 [file ijms-22-04386-s001.zip › ijms-1179164-supplementary.pdf]

Supplementary Materials

Supplementary Figures

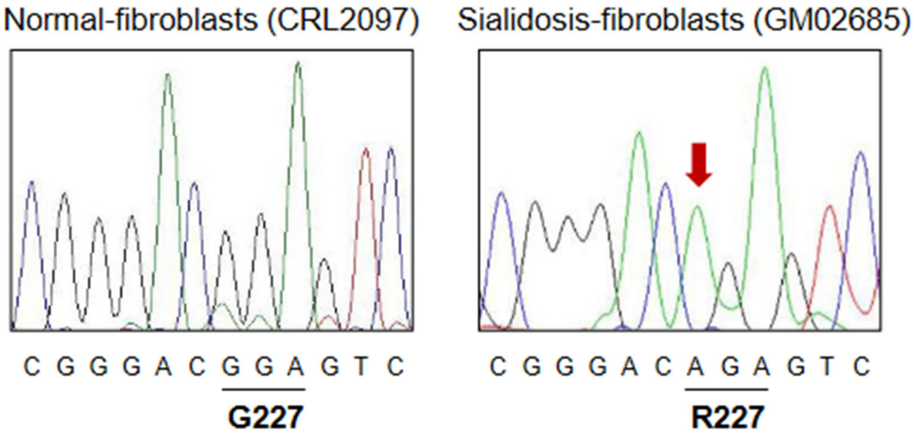

Supplementary Figure S1. Confirmation of DNA sequences of the *NEU1* gene in normal and sialidosis patient fibroblasts carrying the G227R mutation in *NEU1*.

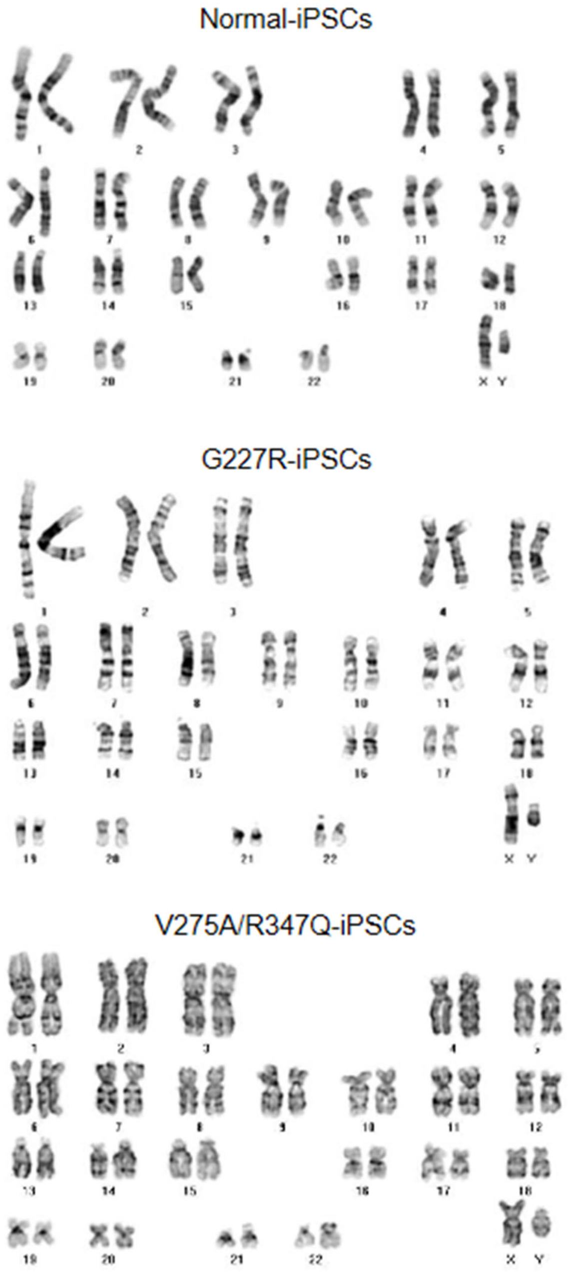

Supplementary Figure S2. Representative G-banding karyotype images of normal- and sialidosis-iPSCs (G227R- and V275A/R347Q-iPSCs) at passages 20–25.

Table 1. STR profiles of normal- and sialidosis-iPSCs and their parental cells.

Clone/Locus	Control				Sialidosis							
	CRL2097 Normal Fibroblasts		Normal-iPSCs		Sialidosis Fibroblast with G227R Mutation		G227R-iPSCs		Sialidosis Fibroblast with V275A/R347Q Mutations		V275A/R347Q-iPSCs	
D8S1179	12	12	12	12	9	9	9	9	10	13	10	13
D21S11	29	31.2	29	31.2	29	30	29	30	30	32.2	30	32.2
D7S820	12	12	12	12	11	11	11	11	10	11	10	11
CSF1PO	12	13	12	13	10	12	10	12	10	12	10	12
D3S1358	15	15	15	15	16	17	16	17	14	18	14	18
TH01	6	9.3	6	9.3	9.3	9.3	9.3	9.3	6	6	6	6
D13S317	11	12	11	12	8	13	8	13	11	12	11	12
D16S539	9	11	9	11	12	12	12	12	11	13	11	13
D2S1338	20	23	20	23	18	20	18	20	16	17	16	17
D19S433	13	15	13	15	14	14	14	14	14	16	14	16
vWA	17	18	17	18	16	18	16	18	16	16	16	16
TPOX	10	11	10	11	8	8	8	8	10	11	10	11
D18S51	13	18	13	18	12	16	12	16	10	22	10	22
D5S818	11	12	11	12	12	12	12	12	11	13	11	13
FGA	24	26	24	26	22.2	24	22.2	24	19	20	19	20
Gender	XY		XY		XY		XY		XY		XY	

Table 2. List of antibodies used in this study.

Antibodies	Target Protein	Company (Cat. No.)	Dilution
Primary antibodies	α-SMA	Sigma (A5228)	1 : 200
	DESMIN	Chemicon (AB907)	1 : 50
	FOXA2	Millipore (07-633)	1 : 100
	GFAP	Abcam (ab134436)	1 : 1000
	LAMP1	Abcam (ab25630)	1 : 1000
	MAP2	Sigma (M1406)	1 : 1000
	NANOG	R&D (AF1997)	1 : 40
	NESTIN	Millipore (MAB5326)	1 : 100
	NEU1	Rockland Immunochemicals (100-401-396)	1 : 100
	O4	R&D (MAB1326)	1 : 100
	Oct3/4	Santa Cruz (sc-9081)	1 : 100
	OLIG-2	Abcam (AB9610)	1 : 500
	PPCA	Abcam (ab184553)	1 : 10000
	SOX17	R&D (MAB1924)	1 : 50
	SSEA-3	R&D (MAB1434)	1 : 30
	SSEA-4	Millipore (MAB1435)	1 : 30
	TRA-1-60	Millipore (MAB4360)	1 : 100
	TRA-1-81	Millipore (MAB4381)	1 : 100
	TUJ1	Biolegend (802001)	1 : 500
Secondary antibodies	Chicken anti-rabbit IgG	Invitrogen (A21442)	1 : 200
	Chicken anti-goat IgG	Invitrogen (A21467)	1 : 200
	Goat anti-mouse IgM	Invitrogen (A21044)	1 : 200
	FITC mouse anti-rat IgM	BD Pharmingen (553887)	1 : 200
	Donkey anti-mouse IgG	Invitrogen (A21202)	1 : 200
	FITC rat anti-mouse IgM	BD Pharmingen (553437)	1 : 200
	Goat anti-rabbit IgG-HRP	Santa Cruz (SC-2004)	1 : 10000
	Goat anti-mouse IgG -HRP	Ab Frontier (LF-SA8001)	1 : 10000
